# Extramedullary AML: Clinical and Molecular Features

**DOI:** 10.3390/cancers18091362

**Published:** 2026-04-24

**Authors:** Yael Morgenstern, Claire Andrews, Eshetu G. Atenafu, Steven Chan, Vikas Gupta, Mark D. Minden, Dawn Maze, Aaron Schimmer, Andre Schuh, Karen Yee, Hassan Sibai

**Affiliations:** 1 Division of Medical Oncology and Hematology, Princess Margaret Cancer Centre, University Health Network, Toronto, ON M5G 1X6, Canada; 2 Department of Biostatistics, University Health Network, Toronto, ON M5G 1X6, Canada; 3 Division of Biostatistics, University of Toronto, Toronto, ON M5S 3E3, Canada; 4 Department of Medicine, Division of Hematology, University of Toronto, Toronto, ON M5S 3H2, Canada

**Keywords:** AML, leukemia, myeloid sarcoma, extra meduallary AML

## Abstract

Background: Acute myeloid leukemia with extramedullary disease (EMD-AML) represents a distinct clinical entity associated with diagnostic and therapeutic challenges, and its prognostic significance remains uncertain. Methods: A retrospective study of 617 adults with newly diagnosed AML (2005–2018) was conducted, analyzing 246 patients with EMD-AML and 371 without EMD involvement. The clinical characteristics and treatment outcomes were analyzed. Propensity score matching (PSM) was applied to adjust for baseline confounders. Results: Patients with isolated EMD-AML and those with concurrent bone marrow involvement had comparable clinical outcomes. NPM1 mutations (48% vs. 25%, *p* = 0.0002) and t(8;21) translocation (23.2% vs. 3.7%, *p* < 0.001) were enriched in the EMD-AML cohort. After PSM, EMD-AML patients achieved a higher overall response rate compared with non-EMD-AML (88.1% vs. 72.0%, *p* = 0.0002) but experienced significantly higher relapse rates (35.7% vs. 15.5%, *p* < 0.0001). Despite the achievement of a higher response rate, EMD-AML was associated with shorter median overall survival (OS) (14.2 vs. 64.1 months, *p* < 0.0001) and event-free survival (EFS) (9.5 vs. 55.9 months, *p* < 0.0001). In a multivariable analysis, EMD-AML remained independently associated with worse OS and EFS (OS HR 1.79, *p* = 0.01; EFS HR 1.95, *p* = 0.001). Allogeneic hematopoietic stem cell transplantation did not confer a survival advantage in EMD-AML patients. Conclusions: EMD-AML, whether isolated or concurrent with bone marrow disease, represents a high-risk entity characterized by poor long-term outcomes despite strong initial response rates. Obtaining tissue biopsies for molecular profiling may help improve risk stratification, identify targetable mutations and guide individualized treatment.

## Simple Summary

Acute myeloid leukemia (AML) with extramedullary involvement occurs in a substantial proportion of newly diagnosed patients. Despite this, there are currently no specific guidelines for its monitoring or treatment, and management is generally extrapolated from conventional AML. In this study, we evaluated the clinical outcomes of patients with extramedullary AML and found that, although they often achieve strong initial responses to treatment, they experience significantly worse overall survival and event-free survival than patients without extramedullary disease. These findings support the view that extramedullary AML represents a biologically and clinically distinct high-risk entity. Our results also highlight the importance of obtaining a biopsy of the extramedullary site, when feasible, together with molecular profiling, to improve risk stratification and identify potential therapeutic targets.

## 1. Introduction

Acute myeloid leukemia (AML) with extramedullary disease (EMD) involvement, also referred to as myeloid sarcoma (MS) is recognized in the current WHO and ICC classification systems as a distinct clinicopathologic entity; however, it often represents an extramedullary manifestation of an underlying AML diagnosis in the bone marrow. It is characterized by the infiltration of clonal myeloid cells into extramedullary sites and may present either as an isolated presentation of AML without involvement of the bone marrow or synchronously with bone marrow disease.

The diagnosis of EMD-AML is challenging and often requires advanced imaging and tissue biopsy. However, biopsies are often deferred given the high level of risk of the procedure in a newly diagnosed AML patient. Reported prevalence rates of EMD-AML vary widely, ranging from 2% to 25% of all AML cases [1,2,3,4,5]. A prospective study utilizing ^18^Fluorodeoxyglucose positron emission tomography/computed tomography (^18^FDG-PET/CT) at diagnosis identified EMD in up to 22% of AML patients. Remarkably, PET/CT findings persisted even in individuals considered to be in complete remission based on bone marrow assessments [5]. In addition, prior reports have suggested that extramedullary relapse may be relatively more frequent following allogeneic hematopoietic stem cell transplantation (allo-HSCT), further supporting the distinct clinical behavior of EMD-AML [6,7]. Further to the diagnostic challenge, recent studies suggest that EMD exhibits distinct genetic and biological features and may also be driven by immunophenotypic mechanisms, including altered HLA expression and impaired antigen presentation, that facilitate immune escape and dissemination to extramedullary sites, highlighting the unique pathophysiology underlying extramedullary AML [2,8,9,10,11,12]. Although EMD-AML has generally been associated with poor outcomes [2,4,13], certain subtypes such as AML with t(8;21) and EMD involvement did not demonstrate a negative prognostic impact [14]. Due to heterogeneity in the clinical presentation, site involvement, molecular profile and response to therapy, EMD is not currently recognized as an independent predictive factor in the European LeukemiaNet (ELN) 2022 risk stratification guidelines.

While EMD-AML is generally considered radiosensitive, radiotherapy (RT) is not routinely incorporated into standard treatment algorithms and is most often used as an adjunct to systemic therapy for rapid local disease control and symptom palliation. In retrospective studies, no survival benefit was seen for the addition of RT to systemic therapy compared to systemic therapy alone [15,16].

Despite advancements in AML treatment, the optimal treatment approach for EMD-AML, including the choice of chemotherapy regimen, the role of allo-HSCT [2], use of RT and the use of targeted therapy [17], remains uncertain. In this study, we aim to evaluate the clinical and molecular characteristics of EMD-AML, with a particular focus on its prognostic significance and the identification of treatment strategies that may confer the greatest benefit.

## 2. Methods

### 2.1. Patient Cohort

**Figure 1:**
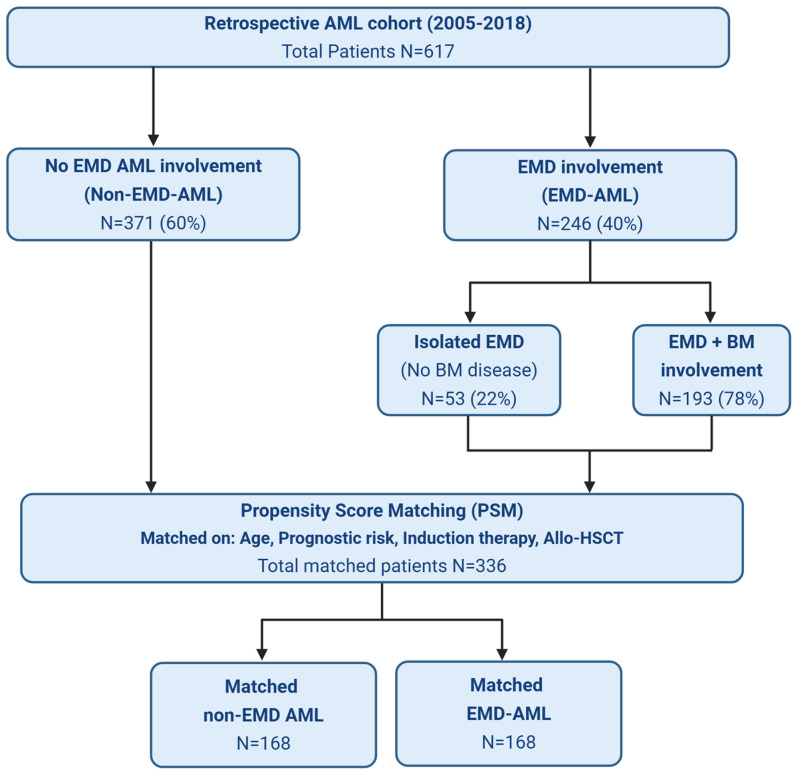
Retrospective cohort study design and study flow diagram of AML patients treated at Princess Margaret cancer center.

We conducted a retrospective study of 617 adults (age ≥ 18 years) with newly diagnosed acute myeloid leukemia (AML) treated at Princess Margaret Cancer Centre (PMCC) between 2005 and 2018. Among these, 246 patients were identified to have EMD-AML disease. The study flow chart is shown in Figure 1. This study was approved by the PMCC Research Ethic Board 19-5397. The clinical, laboratory, and treatment-related data were obtained through systematic chart review.

### 2.2. Statistical Analysis

The categorical variables were summarized using counts and percentages. Continuous variables were summarized using medians with interquartile ranges (IQRs) or ranges, or means with standard deviations, as appropriate based on distribution. Group comparisons were assessed using the Kruskal–Wallis test or *t*-test for continuous variables, and the Chi-square or Fisher’s exact test for categorical variables, as appropriate.

Owing to differences in baseline characteristics between patients with and without EMD, 1:1 propensity score matching (PSM) was applied to reduce confounding and balance covariates between groups. The variables included in the propensity score model were age, prognostic risk category, type of induction therapy, and therapy that included allo-HSCT. NPM1 mutation status was excluded from the PSM model due to a high proportion of missing data, which would have substantially reduced the matched cohort size. PSM was conducted to mitigate the bias typically associated with conventional multivariable adjustment.

Overall survival (OS) and event-free survival (EFS) rates were estimated using the Kaplan–Meier method. Survival curves were compared using the log-rank test to evaluate the impact of EMD at diagnosis. All *p*-values were two-sided, and a *p* < 0.05 was considered statistically significant. All statistical analyses were performed using SAS version 9.4 for Windows (Copyright © 2023 by SAS Institute Inc., Cary, NC, USA).

## 3. Results

### 3.1. Demographics and Clinical Features

**Figure 2:**
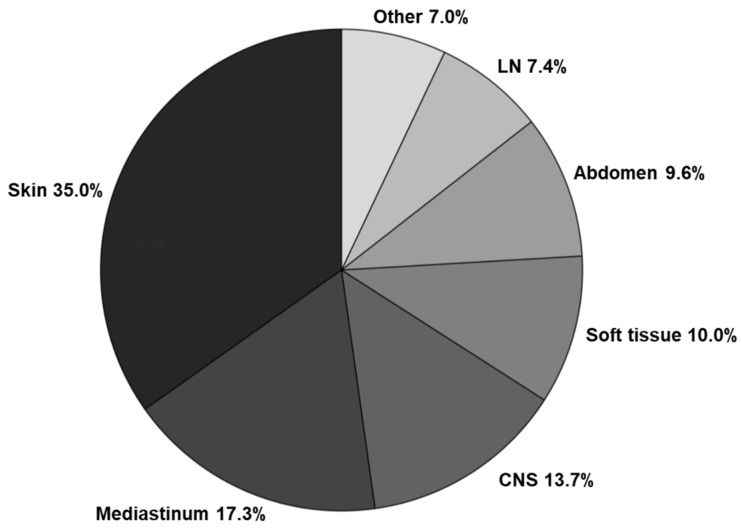
Distribution of extramedullary disease sites in AML.

A total of 617 patients with AML were included in the analysis. Of these, 371 (60%) had no evidence of extramedullary disease (EMD) involvement (no EMD AML), while 246 (40%) presented with EMD (EMD-AML). Among the 246 patients with EMD-AML, 53 (22%) had isolated extramedullary involvement without bone marrow (BM) disease, whereas 193 showed concurrent involvement of both BM and EMD sites. The most frequently involved sites were the skin (38%), mediastinum (19%), CNS (15%), and abdomen (11%) (Figure 2). Most patients had a single site of involvement, while 7.7% showed multiple sites affected. When comparing baseline characteristics (Supplementary Table S1), patients with EMD-AML were significantly younger (median age 57.1 vs. 60.1, *p* = 0.03), had a lower proportion of unfavorable risk attributes (24.4% vs. 59.3%, *p* < 0.0001), were less frequently treated with FLAG-IDA or NOVE-HIDAC (10.6% vs. 26.4%, *p* < 0.001), and fewer proceeded to allo-HSCT (33.9% vs. 43.1%, *p* = 0.02). Importantly, no significant differences in sex, karyotype, mutation profile, or allo-HSCT rates were found between patients with isolated EMD and those with concurrent BM and EMD involvement (Supplementary Table S2). Patients with combined BM involvement and EMD had a higher proportion of favorable risk characteristics and were less frequently treated with FLAG-IDA or NOVE-HIDAC protocols. Since both groups showed no significant differences in ORR, OS, EFS and relapse rate, they were collectively analyzed as a single group, EMD-AML, in subsequent analyses when being compared to the non-EMD-AML cohort (Supplementary Table S2, Supplementary Figure S1).

### 3.2. Molecular and Cytogenetic Analysis

**Figure 3:**
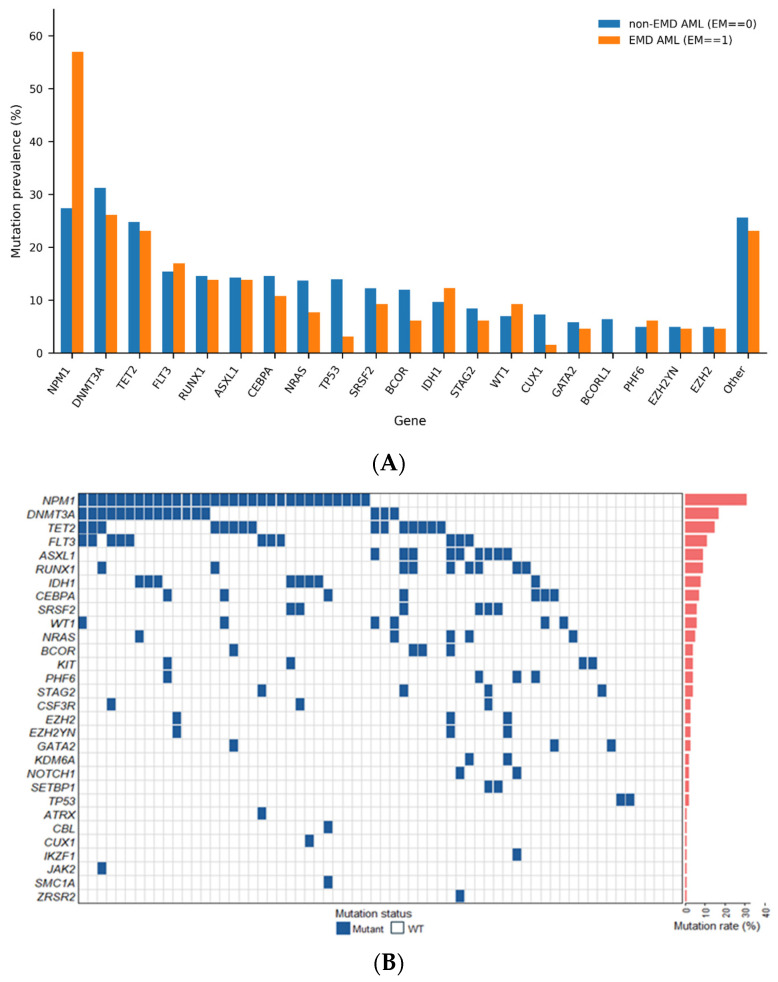
Genomic landscape of AML and EMD-AML. (**A**) Distribution of gene mutations across patients with AML (**B**) Mutational landscape of EMD-AML patients with available NGS from bone marrow. Percentage of patients positive for each of the mutations is marked in red color.

Bone marrow next-generation sequencing (NGS) data were available for patients in the non-EMD-AML cohort (*n* = 371), whereas only 26% (*n* = 64) of patients in the EMD-AML group had available NGS results. Among the patients with available NGS results in the EMD-AML cohort, the most frequently observed mutations were in *NPM1* (48%), *DNMT3A* (27%), *TET2* (23%), and *FLT3* (17%) (Figure 3A,B). Notably, *NPM1* mutations were significantly more common in the EMD-AML group compared to the non-EMD-AML group (48% vs. 25%, *p* = 0.0002). No statistically significant differences were observed in the distribution of other mutations between the two groups. For cytogenetic analysis, results were available for 87% (*n* = 324) of patients in the non-EMD-AML group and 23% (*n* = 56) of those in the EMD-AML group. Among patients with evaluable cytogenetic data, t(8;21) was significantly enriched in the EMD-AML cohort compared with the non-EMD-AML cohort (23.2% vs. 3.7%, *p* < 0.001). In contrast, the frequency of complex karyotype did not differ significantly between groups (4% vs. 12%, *p* = 0.06) (Supplementary Table S1).

At a median follow-up of 19.7 months (range 0.0–250.2) for the entire cohort, ORR was 77.2%, with relapse occurring in 23.3% of patients. Median OS was 24.3 months (95% CI 18.9–33.1–250.2) months, and median EFS was 18.8 months (range 15.1–25.4) months. Patients with EMD-AML exhibited a significantly higher ORR of 90.7% compared to 68.2% in those without EMD involvement (*p* < 0.0001). Despite this improved initial response, EMD-AML patients experienced a higher relapse rate (33.7% vs. 16.4%, *p* < 0.0001), along with shorter median OS of 14.2 months (95% CI 11.8–16.9) vs. 53.2 months (95% CI range 39.8–67.8), *p* < 0.0001, as well as shorter EFS of 10.6 months (95% CI 8.7–13.6) vs. 43.7 months (95% CI 31.1–58.4), *p* < 0.0001, compared to the non-EMD-AML group. Given significant baseline differences between the EMD-AML and non-EMD-AML cohorts, including age, risk stratification, induction regimen, and rates of allogeneic hematopoietic stem cell transplantation (allo-HSCT), a propensity score matching (PSM) analysis was performed to adjust for these confounding variables. After PSM, the baseline characteristics of non-EMD-AML and EMD-AML cohorts matched closely (Table 1). Notably, *NPM1* mutation status was excluded from the propensity score matching analysis because molecular data were incomplete in a substantial proportion of EMD-AML patients. Consequently, the EMD-AML cohort continued to demonstrate a higher frequency of NPM1-mutated AML relative to the non-EMD-AML group even after matching.

**Figure 4:**
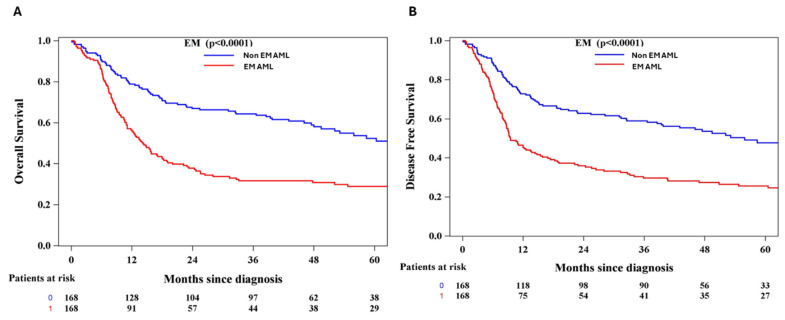
Kaplan–Meier survival curves after propensity score matching. (**A**) Overall survival and (**B**) Disease-free survival curves stratified by presence of EMD.

In the matched cohorts, EMD-AML still showed better response rate compared to the non-EMD-AML group with a median ORR of 88.1% vs. 72.0% (*p* = 0.0002). Nevertheless, even after PSM and despite higher ORR, EMD-AML was associated with significantly lower rates of OS compared to the cohort of non-EMD-AML (median OS 14.2 vs. 64.1 months, 2-year OS 37.8% vs. 67.0%, *p* < 0.0001), and lower EFS (median EFS 9.5 vs. 55.9 months, 2-year EFS 36.0% vs. 62.8%, *p* < 0.0001) (Figure 4A,B). In a multivariable adjusted (MVA) model, presence of EMD-AML remained an independent risk factor of inferior OS and EFS, underscoring its potential biological and clinical importance for OS (HR 1.79 [95%–CI: 1.22–2.62], *p* = 0.0099) and EFS (HR 1.95 [95%–CI: 1.36–2.79], *p* = 0.001) (Table 2 and Table 3).

### 3.3. Outcomes in ELN Favorable-Risk Subgroup

Given the association between EMD-AML and inferior OS and EFS observed in our cohort, we further evaluated its predictive impact among patients classified within the ELN favorable-risk group. In the PSM-matched cohort, ELN favorable-risk patients with EMD-AML demonstrated a trend toward poorer outcomes compared with their favorable-risk non-EMD-AML counterparts. The 2-year EFS rate was marginally lower in the EMD-AML group at 52.0% (95% CI, 35.0–66.5) versus 73.8% (95% CI, 57.7–84.6) in the non-EMD-AML group (*p* = 0.053). Correspondingly, a trend toward higher cumulative incidence of relapse was observed among patients with EMD-AML (30.8% vs. 13.6%, *p* = 0.059). The 2-year OS rates showed a comparable trend, with 55.8% (95% CI, 38.1–70.2) for EMD-AML versus 75.9% (95% CI, 59.8–86.3) for non-EMD-AML. However, this difference did not reach statistical significance (*p* = 0.081; Table 4).

### 3.4. Analysis of Prognostic Factors Within EMD-AML

To further explore factors associated with survival in the EMD-AML cohort, MVA was performed for OS and EFS. Within the EMD-AML subgroup, several variables were evaluated but did not demonstrate significant associations with OS or EFS, including the presence of isolated EMD without bone marrow involvement, prognosis risk category, type of induction therapy, presence of NPM1 mutation, and age.

Notably, among the 246 patients with EMD-AML, 83 patients (33.7%) underwent allogeneic hematopoietic stem cell transplantation (allo-HSCT). In this subgroup, no significant survival benefit was observed for patients who proceeded to allo-HSCT, with OS showing an HR of 0.71 (95% CI 0.34–1.49, *p* = 0.3685) and EFS showing an HR of 0.57 (95% CI 0.29–1.10, *p* = 0.0952) (Table 5 and Table 6).

## 4. Discussion

EMD occurs in up to 25% of patients with AML but is often underrecognized in routine practice [5]. Another diagnostic challenge is the potential for immunophenotypic overlap with other hematological malignancies, as myeloid sarcoma can occasionally express aberrant B- or T-cell markers, increasing the risk of misclassification in isolated extramedullary lesions [18,19,20]. Standard treatment typically consists of intensive chemotherapy, with or without local irradiation [21]; however, the long-term implications of EMD remain uncertain [2,13,14,22,23,24,25,26,27,28,29] and are not currently incorporated into ELN risk stratification. This gap, combined with the frequent exclusion of EMD-AML patients with limited involvement of bone marrow from clinical trials, has limited the evidence base to guide optimal management strategies.

In this study, we evaluated clinical outcomes of patients with EMD-AML, including those presenting with isolated extramedullary involvement and those with concurrent bone marrow disease. We found that although patients with EMD-AML achieved higher ORR than non-EMD-AML patients, they experienced significantly inferior long-term outcomes, with reduced OS and EFS. Importantly, outcomes were similar between patients with isolated EMD and those with concomitant bone marrow involvement, suggesting that both presentations represent biologically comparable high-risk disease and should be managed similarly. Notably, the adverse prognostic impact of EMD extended even to the favorable-risk group, in whom EMD was associated with lower EFS compared to non-EMD patients. The paradox of superior initial response yet poorer long-term outcomes may reflect limitations in current imaging modalities and response assessment tools, which may underestimate residual or refractory disease at extramedullary sites. With advances in cell-free DNA (cfDNA) analysis, plasma cfDNA-based assays may offer a more sensitive approach to response assessment, minimal residual disease monitoring, and early relapse detection in EMD-AML. In support of this, a recent study showed that cfDNA enabled highly sensitive MRD surveillance among EMD-AML patients, with detection of 83% of relapse cases [30].

The role of allogeneic transplantation in this setting remains controversial: some studies have suggested a potential survival benefit, while others have not shown clear improvement in outcomes for EMD-AML [31,32,33]. Furthermore, relapse involving extramedullary sites is more frequently observed in the post-allo-HSCT setting [34]. Our results suggest that allogeneic HSCT does not overcome the adverse prognosis associated with EMD. These findings further emphasize the need for prospective studies to identify the optimal therapeutic approach for this high-risk population.

Molecular characterization of EMD-AML in our cohort was limited by the availability of bone marrow NGS data, available for only 26% of affected patients, compared with a substantially larger proportion of the non-EMD-AML cohort. Given the more limited molecular data in the EMD-AML group compared with the non-EMD-AML cohort, the genomic findings should be interpreted with appropriate caution. Within these constraints, the mutational landscape was broadly similar between EMD-AML and non-EMD-AML, except for a higher frequency of NPM1 mutations in the EMD cohort. Interestingly, despite the established association of NPM1 mutations with favorable prognosis in AML without extramedullary involvement, our MVA did not demonstrate an OS or EFS benefit for NPM1-mutated patients within the EMD-AML cohort. This suggests that the prognostic advantage typically attributed to NPM1 may be attenuated or lost in the presence of extramedullary disease.

Prior studies suggest that EMD may harbor distinct biological and molecular features [8,9,10,11,12,35,36,37]. Furthermore, the frequent occurrence of EMD as a manifestation of relapse in the post-allogeneic HSCT setting further supports the distinct biology of this entity [6]. Despite these observations, routine molecular profiling of extramedullary sites is rarely performed, potentially leading to under-recognition of genetically distinct or actionable lesions.

This study has several limitations, including its retrospective design, limited NGS data for the EMD-AML cohort, incomplete evaluation of site-specific treatment responses, and the absence of patients receiving contemporary low-intensity or targeted therapies such as hypomethylating agents with venetoclax or mutation-directed agents [17,38,39,40]. In addition, immunohistochemical and flow cytometric immunophenotypic data were not systematically available, limiting our ability to further characterize the biological heterogeneity of extramedullary lesions, including potential myeloid versus monoblastic differentiation patterns.

In conclusion, our findings support the classification of EMD-AML, regardless of isolated or concurrent bone marrow involvement, as a high-risk entity associated with inferior outcomes. Although allogeneic HSCT is often pursued due to high relapse risk, our data suggest that transplant alone does not mitigate the poor prognosis of these patients. While skin biopsies of extramedullary lesions are relatively easy to obtain in newly diagnosed patients, they may yield false-positive results due to contamination by circulating blasts. These results underscore the importance of obtaining tissue biopsies and performing molecular profiling of extramedullary lesions, which may uncover targetable mutations and guide more individualized therapeutic strategies. Prospective studies are essential to improve outcomes for this challenging AML subset.

## 5. Conclusions

EMD-AML demonstrates a distinct clinical course compared with AML without extramedullary involvement and is associated with inferior overall survival and event-free survival. Our findings support the consideration of EMD-AML as a high-risk disease entity and underscore the value of obtaining biopsy material from extramedullary sites, when feasible, for molecular profiling to identify potentially targetable alterations.

## Figures and Tables

**Table 1:** Baseline patient characteristics after PSM.

	Total (*N* = 336)	Non-EMD-AML (*N* = 168)	EMD-AML (*N* = 168)	*p*-Value
Age at diagnosis in years				0.31
Median (IQR)	56.05 (42.95, 64.55)	57.60 (44.90, 65.35)	55.50 (42.55, 63.40)	
Range	18.00, 83.00	18.00, 78.20	18.20, 83.00	
Sex, *n* (%)				0.01
Female	148 (44.05%)	86 (51.19%)	62 (36.90%)	
Male	188 (55.95%)	82 (48.81%)	106 (63.10%)	
Prognosis, *n* (%)				0.66
Favorable	83 (24.70%)	44 (26.19%)	39 (23.21%)	
Intermediate	142 (42.26%)	67 (39.88%)	75 (44.64%)	
Unfavorable	111 (33.04%)	57 (33.93%)	54 (32.14%)	
NPM1, *n* (%)				0.04
Mutated	78 (34.21%)	51 (30.36%)	27 (45.00%)	
Missing	108	0	108	
FLT3, *n* (%)				0.12
Mutated	42 (18.42%)	35 (20.83%)	7 (11.67%)	
Missing	108	0	108	
TET2, *n* (%)				0.71
Mutated	53 (23.25%)	38 (22.62%)	15 (25.00%)	
Missing	108	0	108	
ASXL1, *n* (%)				0.05
Mutated	20 (8.77%)	11 (6.55%)	9 (15.00%)	
Missing	108	0	108	
DNMT3A, *n* (%)				0.54
Mutated	60 (26.32%)	46 (27.38%)	14 (23.33%)	
Missing	108	0	108	
CK, *n* (%)				1.00
Yes	8 (4.04%)	6 (4.14%)	2 (3.77%)	
Missing	138	23	115	
Type of Induction, *n* (%)				0.63
3 + 7	291 (86.61%)	144 (85.71%)	147 (87.50%)	
FLAG-IDA/NOVE-HIDAC	45 (13.39%)	24 (14.29%)	21 (12.50%)	
ORR, *n* (%)	269 (80.06%)	121 (72.02%)	148 (88.10%)	0.0002
Relapse, *n* (%)	86 (25.60%)	26 (15.48%)	60 (35.71%)	<0.0001
Allo-HSCT, *n* (%)	117 (34.82%)	64 (38.10%)	53 (31.55%)	0.21
Isolated EMD, *n* (%)				
Yes	30 (17.86%)	0 (.%)	30 (17.86%)	
Missing	168	168	0	

**Table 2:** Multivariable cox regression analyses for OS.

Parameter	HR (95% CI)	*p*-Value
EMD involvement		0.010
Non-EMD-AML	1.00 (Ref)	
Isolated EMD-AML	1.63 (0.39–6.75)	
EMD-AML with BM involvement	1.79 (1.22–2.62)	
Prognosis category		0.003
Favorable	1.00 (Ref)	
Intermediate	1.81 (1.12–2.94)	0.016
Unfavorable	2.06 (1.36–3.12)	0.001
NPM1 mutation	0.53 (0.36–0.77)	0.001
Type of Induction		0.011
3 + 7	1.00 (Ref)	
FLAG-IDA/NOVE-HIDAC	1.47 (1.09–1.96)	
Allo-HSCT	0.58 (0.44–0.77)	<0.001
Age	1.02 (1.01–1.03)	<0.001

**Table 3:** Multivariable cox regression analyses for EFS.

Parameter	HR (95% CI)	*p*-Value
EMD involvement		0.001
Non-EMD-AML	1.00 (Ref)	
Isolated EMD-AML	1.74 (0.42–7.22)	
EMD-AML with BM involvement	1.95 (1.36–2.79)	
Prognosis category		0.011
Favorable	1.00 (Ref)	
Intermediate	1.75 (1.12–2.74)	
Unfavorable	1.78 (1.21–2.62)	
NPM1 mutation	0.48 (0.33- 0.68)	<0.001
Type of induction		0.030
3 + 7	1.00 (Ref)	
FLAG-IDA/NOVE-HIDAC	1.38 (1.03–1.84)	
Allo-HSCT	0.56 (0.43–0.73)	<0.001
Age	1.02 (1.01–1.03)	<0.001

**Table 4:** Outcomes of favorable risk group after PSM.

	Total (*N* = 83)	Non-EMD-AML (*N* = 44)	EMD-AML (*N* = 39)	*p*-Value
Allo, *n* (%)	21 (25.30%)	8 (18.18%)	13 (33.33%)	0.113
Relapse, *n* (%)	18 (21.69%)	6 (13.64%)	12 (30.77%)	0.059
ORR, *n* (%)	69 (83.13%)	36 (81.82%)	33 (84.62%)	0.734
2-year EFS rate (95% CI)		73.8% (57.7–84.6)	52% (35.0–66.5)	0.053
2-year OS rate (95% CI)		75.9% (59.8–86.3)	55.8% (38.1–70.2)	0.081

**Table 5:** Multivariable cox regression analyses for OS in EMD-AML.

Parameter	Frequency	HR (95% CI)	*p*-Value
EMD-AML with BM involvement	193 vs. 53	1.12 (0.19–6.49)	0.899
Allo-HSCT	83 vs. 162	0.71 (0.34–1.49)	0.369
Type of Induction			0.299
3 + 7	186	1.00 (Ref)	
FLAG-IDA/NOVE-HIDAC	22	1.70 (0.62–4.65)	
Prognosis category			0.235
Favorable	39	1.00 (Ref)	
Intermediate	147	0.99 (0.30–3.30)	
Unfavorable	60	1.92 (0.84–4.41)	
Age	246	1.02 (0.99–1.06)	0.171
NPM1 (%)	31 vs. 33	0.72 (0.33–1.58)	0.415

**Table 6:** Multivariable cox regression analyses for EFS in EMD-AML.

Parameter	Frequency	HR (95% CI)	*p*-Value
EMD-AML with BM involvement	193 vs. 53	0.92 (0.16–5.38)	0.925
Allo-HSCT	83 vs. 162	0.57 (0.29–1.10)	0.095
Type of Induction			0.367
3 + 7	186	1.00 (Ref)	
FLAG-IDA/NOVE-HIDAC	22	1.57 (0.59–4.23)	
Prognosis category			0.628
Favorable	39	1.00 (Ref)	
Intermediate	147	1.50 (0.55–4.08)	
Unfavorable	60	1.37 (0.65–2.90)	
Age	246	1.02 (0.99–1.05)	0.117
NPM1 (%)	31 vs. 33	0.53 (0.25–1.11)	0.091

## Data Availability

The study data and materials are available from the senior author Hassan Sibai.

## Supplementary Materials

The following supporting information can be downloaded at https://www.mdpi.com/article/10.3390/cancers18091362/s1, Figure S1: OS and EFS Kaplan–Meier survival curves of EMD-AML patients stratified by type of EMD as isolated versus presentation with concurrent bone marrow disease; Table S1: baseline patient characteristics before PSM; Table S2: baseline clinical characteristics of EMD-AML patients.
