# Supplementary material for: Extramedullary AML: Clinical and Molecular Features
Source: Cancers (Basel). 2026 Apr 24;18(9):1362. doi: 10.3390/cancers18091362 (PMC13162922; doi:10.3390/cancers18091362)
Supplement: Supplementary file 1 [file cancers-18-01362-s001.zip › cancers-4248379-supplementary.pdf]

Supplementary Figure 1

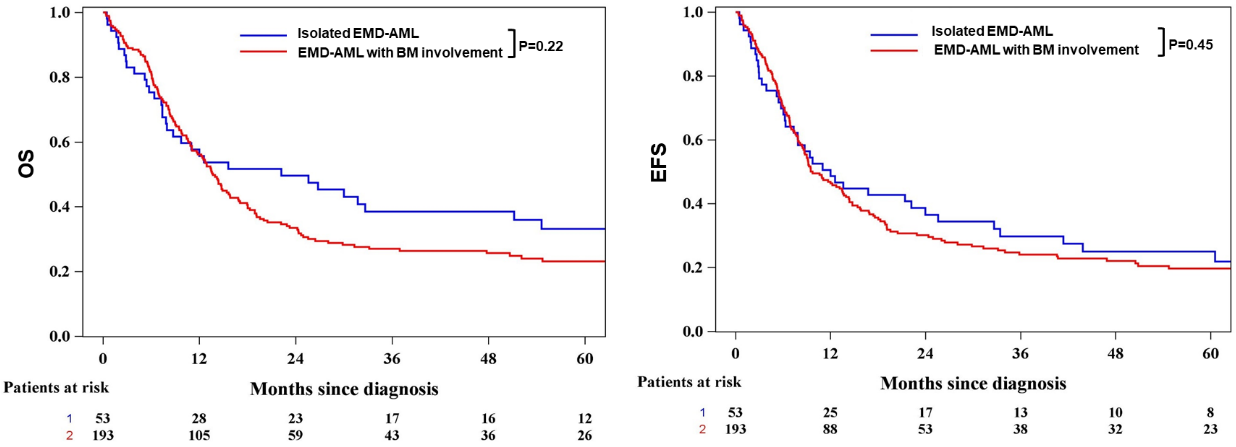

Kaplan Meier survival curves of EMD-AML patients. (A) OS and (B) EFS for patients with EMD-AML stratified by type of EMD as isolated versus presentation with concurrent bone marrow disease. Abbreviations: OS, overall survival; EFS, event free survival

Supplementary table 1 Baseline patient characteristics

	Total (N=617)	Non-EMD AML (N=371)	EMD-AML (N=246)	P-value
**Isolated EMD, n (%)**	53 (21.54%)	0 (.%)	53 (21.54%)	
**Age (years), median (IQR)**	59.2 (47-68)	60.1 (51-68)	57.1 (45-67	**0.03**
**Sex, n (%)**				0.13
**Female**	274 (44.41%)	174 (46.90%)	100 (40.65%)	
**Male**	343 (55.59%)	197 (53.10%)	146 (59.35%)	
**ELN risk, n (%)**				**<.0001**
**Favourable**	121 (19.61%)	82 (22.10%)	39 (15.85%)	
**Intermediate**	216 (35.01%)	69 (18.60%)	147 (59.76%)	
**Unfavourable**	280 (45.38%)	220 (59.30%)	60 (24.39%)	
**Evaluable patients with mutation testing, n**	435	371	64	
**Mutations n (%)**				
**NPM1**	125 (28.74%)	94 (25.34%)	31 (48.44%)	**0.0002**
**FLT3, n (%)**	64 (14.71%)	53 (14.29%)	11 (17.19%)	0.55
**TET2, n (%)**	100 (22.99%)	85 (22.91%)	15 (23.44%)	0.93
**ASXL1, n (%)**	58 (13.33%)	49 (13.21%)	9 (14.06%)	0.85
**DNMT3A, n (%)**	124 (28.51%)	107 (28.84%)	17 (26.56%)	0.71
**Cytogenetics n (%)**				
**Complex karyotype**	41 (10.79%)	39 (12.04%)	2 (3.57%)	0.06
**t(8;21)**	25 (6.6%)	12 (3.7%)	13 (23.2%)	**<.0001**
**inv(16) or t(6;16)**	16 (4.2%)	15 (4.6%)	1 (1.8%)	0.33
**Not available**	237	47	190	
**Type of Induction, n (%)**				**<.0001**
**3+7**	459 (79.27%)	273 (73.58%)	186 (89.42%)	
**FLAG-IDA/NOVE-HIDAC**	120 (20.73%)	98 (26.42%)	22 (10.58%)	
**Other**	38	0	38	
**ORR, n (%)**	476 (77.15%)	253 (68.19%)	223 (90.65%)	**<.0001**
**Relapse, n (%)**	144 (23.34%)	61 (16.44%)	83 (33.74%)	**<.0001**
**Allo, n (%)**	241 (39.38%)	158 (43.05%)	83 (33.88%)	0.02

Supplementary table 2 Baseline clinical characteristics of EMD-AML patients

	Group		
	**Isolated EMD (N=53)**	**Concurrent BM+EMD (N=193)**	**P-value**
**Age at diagnosis in years**			0.98
**N**	53	193	
**Median (IQR)**	55.80 (46.10, 66.50)	57.30 (44.80, 67.30)	
**Sex, n (%)**			0.65
**Female**	23 (43.40%)	77 (39.90%)	
**Male**	30 (56.60%)	116 (60.10%)	
**Prognosis, n (%)**			<.0001
**Favourable**	1 (1.89%)	38 (19.69%)	
**Intermediate**	46 (86.79%)	101 (52.33%)	
**Unfavourable**	6 (11.32%)	54 (27.98%)	
**NPM1, n (%)**			0.49
**Not Mutated**	2 (100.00%)	31 (50.00%)	
**Mutated**	0 (0.00%)	31 (50.00%)	
**Not available**	51	131	
**FLT3, n (%)**			1.00
**Not Mutated**	2 (100.00%)	51 (82.26%)	
**Mutated**	0 (0.00%)	11 (17.74%)	
**Not available**	51	131	
**TET2, n (%)**			0.42
**Not Mutated**	1 (50.00%)	48 (77.42%)	
**Mutated**	1 (50.00%)	14 (22.58%)	
**Not available**	51	131	
**ASXL1, n (%)**			0.26
**Not Mutated**	1 (50.00%)	54 (87.10%)	
**Mutated**	1 (50.00%)	8 (12.90%)	
**Not available**	51	131	
**DNMT3A, n (%)**			0.46
**Not Mutated**	1 (50.00%)	46 (74.19%)	
**Mutated**	1 (50.00%)	16 (25.81%)	
**Not available**	51	131	
**CK, n (%)**			1.00
**Yes**	0 (0.00%)	2 (3.70%)	
**Not available**	51	139	
**Type of Induction, n (%)**			0.008
**3+7**	32 (78.05%)	154 (92.22%)	
**FLAG-IDA/NOVE-HIDAC**	9 (21.95%)	13 (7.78%)	
**Other**	12	26	

**ORR n (%)**	51 (96.23%)	172 (89.12%)	0.18
**Relapse, n (%)**	17 (32.08%)	66 (34.20%)	0.77
**Allo, n (%)**	21 (39.62%)	62 (32.29%)	0.32
